# Remedies and Their Uses

**Published:** 1906-12-29

**Authors:** 


### Remedies and their Uses.

### Scopolamine-Morphine.

Scopolamine hydrobromide of commerce is not a pure alkaloidal salt, but contains two alkaloidal bodies, hyoscine and atroscine, in varying proportions. Hyoscine and atroscine are both narcotics with a marked mydriàtic action. It has recently been used together with morphine hypodermically to produce general anæsthesia. Its action is somewhat variable (owing no doubt to the fact that it consists of two substances), and it does not keep well, but in spite of these two facts it has been somewhat extensively employed and found satisfactory, mainly as a preliminary to chloroform anæsthesia. It was introduced by Desjardine in 1904. Terrier [1] reported 26 operations in 1905, Lorenzelli [2] 200 in 1906. Buchanan,[3] in Glasgow, has published an account of 4 cases; and Schmidt,[4] in May 1905, made scopolamine-morphine narcosis the subject of an inaugural dissertation at Freiberg. The usual procedure is to give 1 centigram morphine and 1 milligram of scopolamine two hours before the operation, a second, and, if necessary, a third dose being given. The eyes should be bandaged and the ears plugged with cotton-wool, or the patient may be left absolutely quiet in the dark. The first effect is general cutaneous vaso-dilatation and mydriasis, the bulbar conjunctiva becoming markedly congested. The patient falls into a heavy sleep and there is considerable analgesia; the third injection is not felt; but the narcosis is not complete and there is no abolition of the reflexes. In four of Lorenzelli's cases there was sufficiently deep narcosis for operative purposes. As a rule, however, a few drops of chloroform are administered to secure complete narcosis with relaxation of the muscles. A very little suffices. For several hours after the operation the patient continues to sleep, and even after he awakes there persists sufficient analgesia to prevent any pain being felt at the site of operation. There may be a little vómiting, but as a rule there is not even nausea. Headache is absent, but there may be considerable thirst, so that it is advisable to inject some normal saline at the conclusion of the operation. Food may be given a few hours after the patient awakens. The injection of scopolamine-morphine may be safely made in children from ten to fifteen years old, 1½ to 2 out of the usual 3 injections being given, according to age. For adults the totals should not exceed 4.5 centigrams of morphia and 3.5 milligrams of scopolamine. These large doses are not necessary if chloroform is to be administered. Owing to the almost total suppression of the period of excitement, it is specially useful in alcoholic subjects. Very little chloroform need ever be given. After the operation the patient is spared the pain and distress usually felt during the first 24 hours. There is no albuminuria or bronchial irritation. The main disadvantages are the general vaso-dilatation which contra-indicates scopolamine where very vascular parts have to be incised. Moreover, the absence of complete muscular relaxation is a drawback unless $CHCl_3$ is given. In abdominal sections only one injection should be given, and in cardiac disease and in alcoholism, the injections must not be pushed after sleep has begun. Owing to the variations in the composition of the drug and in the idiosyncrasy of patients, $CHCl_3$ must always be in readiness. The solutions must, moreover, be freshly prepared. Apomorphine is said to occur in solutions of scopolamine and morphine which have been kept any time. The general conclusions of Roith,[5] who has reviewed the subject as a whole, are in agreement with those of other authors. From the experience at Heidelberg he concludes that scopolamine-morphine injections are perfectly safe in the small doses advocated above, given at the proper intervals. No special experience is necessary if the general rules are carefully carried out, and there are no unpleasant after-effects.

Laurendeau,[6] after trying scopolamine-morphine anæsthesia for a case of symphysiotomy, was so satisfied with the result that he adopted the same plan for ordinary midwifery practice. He gave one to three injections of $\frac{1}{50}$ grain scopolamine and $\frac{1}{5}$ grain morphine at intervals of six hours. By this means he induced a sleep lasting for from 12 to 18 hours. He found that there were no unfavourable symptoms for the mother, but that in some cases the child was slightly asphyxiated at birth. In all his cases either forceps or version was necessary, and in the majority a little chloroform was also given. The main physiological effect appeared to him to be on the respiratory centre, the respirations being slower and deeper. The heart was much less affected, but there was some quickening of the pulse-rate.

Penkert [7] has combined scopolamine-morphine injections with spinal anæsthesia. Altogether he has adopted this method in 140 gynæcological cases, 94 of which were major operations. His object was to avoid the nervous disturbance so often occurring in women under spinal anæsthesia, owing to their being able to watch all the preparations, and even the operation itself. The patients were prepared for operation and the skin disinfected on the previous evening, and 2½ to 3 hours before the operation they were given .0003 gram scopolamine and .001 gram morphine hypodermically, the dose being repeated once or twice at intervals of one hour. The patients were protected from light and noise in the usual way. A condition of semi-narcosis was then induced, and immediately before the operation stovaine and supra-renalin were injected by Bier's method between the first and second lumbar vertebræ, 1½ centimetre outside the middle line. A little cerebro-spinal fluid was drawn into the syringe and mixed with the solution which was then slowly injected into the spinal canal. The puncture was never painful, and complete anæsthesia was produced in from 5 to 7 minutes. There were no unpleasant incidents except slight nausea, and in 4 cases somewhat severe post-operative headache, lasting for four days.

[1] Bull et Mem. de la Soc. de Chir. de Paris, No. 6, 1905; Brit. Med. Jour. Epit., March 25, 1905, No. 186. [2] Reforma Med., No. 7, 1906. [3] Glasgow Med. Jour., April 1906. [4] M. M. W., May 1906, p. 1095. [5] Ibid., lii. 46, 1905. [6] Presse Méd., 93, p. 750, 1905. [7] M. M. W., April 3, 1906.

